# Bexarotene Gel: A New Topical Therapy for Alopecia Areata

**DOI:** 10.4103/0974-7753.66921

**Published:** 2010

**Authors:** Mahajan Rajiv, NR Singh

**Affiliations:** Department of Pharmacology, Adesh Institute of Medical Sciences and Research, Bathinda, Punjab, India; 1 Department of Pharmacology, Government Medical College, Amritsar, Punjab, India

Alopecia areata (AA) is an autoimmune disease characterized by discrete patches of nonscarring hair loss. Because it mostly causes bald spots on the scalp, especially in the first stages, it is also called spot baldness. In 1—2% of cases, the condition can spread to the entire scalp (alopecia totalis) or to the entire epidermis (alopecia universalis). The pathogenesis of AA remains enigmatic, but the hair loss is triggered by perifollicular and intrafollicular mononuclear cell infiltrates, composed primarily of activated CD4+ and CD8+ T cells.[1] AA often remits spontaneously, but lymphosuppressive and lymphotoxic treatments such as oral and intralesional corticosteroids facilitate hair regrowth in upto 60% of cases.[2] Systemic steroids have even been combined with 2% topical minoxidil for its treatment.[3] However, oral corticosteroid use is limited by systemic toxicity, while intralesional corticosteroids are difficult to administer to large areas and may induce local skin atrophy.

Bexarotene is a member of a subclass of retinoids that selectively activate retinoid X receptors (RXRs). The chemical name is 4-[1-(5,6,7,8-tetrahydro-3,5,5,8,8-pentamethyl-2-naphthalenyl)ethenyl] benzoic acid.[4] Orally, it has been approved for the treatment of refractory cutaneous T-cell lymphomas (CTCL).[5] Bexarotene gel 1% is indicated for the topical treatment of cutaneous lesions in patients with refractory or persistent CTCL (Stage IA and IB) or who have not tolerated other therapies.[4] Bexarotene gel has also been found to be effective in mycosis fungoides and lymphomatoid papulosis lesions refractory to oral bexarotene and denileukin diftitox as adjuvant therapy.[6] It was also noted that topical bexarotene yielded significant hair regrowth when used to treat patients with follicular mucinosis or folliculotropic mycosis fungoides, and thus it was theorized that topical bexarotene may also induce hair regrowth in AA.[7]

Recently, Talpur and colleagues conducted a prospective ‘half-head’ trial of 1% bexarotene gel, applied twice daily to areas of AA for up to 6 months. They enrolled 42 patients with patchy AA (*n* = 34), alopecia totalis (*n* = 3), and alopecia universalis (*n* = 5). Patients who experienced greater than 50% improvement were considered to be responders. In addition, signs of toxicity were assessed at multiple time points. Patients who responded to the first 6 months of treatment could apply bexarotene gel to both sides of their scalp for an additional 6 months.[8]

During the 6-month half-head treatment phase, the investigators noted that five patients (12%) showed at least 50% hair regrowth on the treated side; six patients (14%) showed at least 50% regrowth on both treated and nontreated sides (postulated due to diffusion of gel or due to noncompliance to protocol) and the treatment was well tolerated. One patient with alopecia universalis showed no hair regrowth during the 5 months of bexarotene gel application; however, when he was started with oral prednisone, he developed significant regrowth only on bexarotene pretreated half of the scalp.[8]

Although AA does not affect other organ systems, nevertheless, patients with AA experience significant distress from this condition, especially in cases of widespread hair loss. Treatment remains a challenge because the most effective options (pulse corticosteroids, oral cyclosporine) carry significant risks. Although the cost of treatment is a limitation with bexarotene gel as 60 g tube of bexarotene gel costs $ 1350, nevertheless, due to significant risk potential of prevailing treatment options a new topical therapy for AA is welcome.
